# Prediction of Multiple Organ Failure Complicated by Moderately Severe or Severe Acute Pancreatitis Based on Machine Learning: A Multicenter Cohort Study

**DOI:** 10.1155/2021/5525118

**Published:** 2021-05-03

**Authors:** Fumin Xu, Xiao Chen, Chenwenya Li, Jing Liu, Qiu Qiu, Mi He, Jingjing Xiao, Zhihui Liu, Bingjun Ji, Dongfeng Chen, Kaijun Liu

**Affiliations:** ^1^Department of Gastroenterology, Daping Hospital, Army Medical University, Chongqing 400042, China; ^2^Department of Nuclear Medicine, Daping Hospital, Army Medical University, Chongqing 400042, China; ^3^School of Basic Medical Sciences, Army Medical University, Chongqing 400038, China; ^4^College of Biomedical Engineering and Imaging Medicine, Army Medical University, Chongqing 400038, China; ^5^Department of Gastroenterology, People's Hospital of Chongqing Hechuan, Chongqing 401520, China; ^6^Department of Medical Engineering, Xinqiao Hospital, Army Medical University, Chongqing 400038, China; ^7^Radiotherapy Center, Sunshine Union Hospital, Weifang, Shandong 261061, China; ^8^Imaging Center, Sunshine Union Hospital, Weifang, 261061 Shandong, China

## Abstract

**Background:**

Multiple organ failure (MOF) may lead to an increased mortality rate of moderately severe (MSAP) or severe acute pancreatitis (SAP). This study is aimed to use machine learning to predict the risk of MOF in the course of disease.

**Methods:**

Clinical and laboratory features with significant differences between patients with and without MOF were screened out by univariate analysis. Prediction models were developed for selected features through six machine learning methods. The models were internally validated with a five-fold cross-validation, and a series of optimal feature subsets were generated in corresponding models. A test set was used to evaluate the predictive performance of the six models.

**Results:**

305 (68%) of 455 patients with MSAP or SAP developed MOF. Eighteen features with significant differences between the group with MOF and without it in the training and validation set were used for modeling. Interleukin-6 levels, creatinine levels, and the kinetic time were the three most important features in the optimal feature subsets selected by K-fold cross-validation. The adaptive boosting algorithm (AdaBoost) showed the best predictive performance with the highest AUC value (0.826; 95% confidence interval: 0.740 to 0.888). The sensitivity of AdaBoost (80.49%) and specificity of logistic regression analysis (93.33%) were the best scores among the six models in the test set.

**Conclusions:**

A predictive model of MOF complicated by MSAP or SAP was successfully developed based on machine learning. The predictive performance was evaluated by a test set, for which AdaBoost showed a satisfactory predictive performance. The study is registered with the China Clinical Trial Registry (Identifier: ChiCTR1800016079).

## 1. Introduction

Acute pancreatitis (AP) is an inflammatory disorder of the pancreas involving local and peripancreatic tissue. Organ failure (OF) is a hallmark complication of severe acute pancreatitis (SAP) and may be found in approximately 20% of all cases of AP [1]. The mortality rate of AP increases as much as 30% when OF occurs [2]. The respiratory, cardiovascular, and renal systems are most frequently involved by AP-induced organ failure [3]. Multiple organ failure (MOF) has a higher mortality rate than OF [2]. Until organ dysfunction occurs, it is difficult to predict the clinical outcome of AP [4]. Therefore, it is crucial to predict the risk of OF at an early phase, so that patients with SAP can be monitored for prompt detection of complications and the need for intensive care [5].

The severity of organ dysfunction in AP can be graded by the modified Marshall grading system [3]. The existing AP scoring systems, such as the Acute Physiology and Chronic Health Evaluation II (APACHE II) and the Ranson score, showed modest value in predicting possible OF. Complicated combinations of predictive methods are more accurate but are not convenient [6]. Therefore, it is important to develop an effective and easily used method to predict the risk of MOF in patients with early AP. Age, comorbid conditions, weight, triglyceride levels, and extent of local pancreatic injury were considered to be risk factors for MOF in patients with AP [7]. Activation of coagulation [8] and levels of cytokines, including interleukin- (IL-) 6 and IL-8 [9, 10], contributed to pancreatic inflammation and systemic injury.

Machine learning (ML), aiming at coping with the unique computational challenges of building statistical models from massive data sets, is a research field at the intersection of statistics and computer science [11]. Artificial intelligence (AI) is a concept to describe subspecialties of computer science such as machine learning, statistical learning, deep learning, and cognitive computing [12, 13]. ML, considered as a subset of artificial intelligence, was not only applied in text mining and classification in the field of computer science [14, 15] but also widely used in clinical practice. In a study by Kim et al. [16], authors developed an artificial intelligence algorithm by using structured data and unstructured clinical notes to predict and diagnose sepsis, which achieved high predictive accuracy 12 hours before the onset of sepsis. Zhang et al. [17] reported outcomes from the latest studies on the management of acute respiratory distress syndrome (ARDS) patients by using an AI algorithm to improve the prediction of the prognosis and care quality. Our previous research preliminarily developed models of machine learning to predict MOF in patients with AP [18]. In this study, we made use of other machine learning algorithms to develop predictive models. The number of included participants was increased, and each model was tested in a prospective cohort of AP patients.

## 2. Methods

### 2.1. Participants

A retrospective analysis was performed in the three affiliated hospitals (Daping Hospital, Southwest Hospital, and Xinqiao Hospital) of the Army Medical University in Chongqing, China, from July 2014 to December 2019. The dataset gathered from patients from July 2014 to May 2018 was regarded as the training and validation set and was retrospectively collected, and the dataset gathered from patients from June 2018 to December 2019 was prospectively recorded as the test set.

The diagnostic criteria for AP were set up according to the revised Atlanta classification of acute pancreatitis 2012. At least two of the following three criteria had to be satisfied for a diagnosis of AP: [1] abdominal pain, [2] serum amylase and/or lipase levels elevated to at least three times the normal upper limit, and [3] characteristic findings of AP on contrast-enhanced computerized tomography, magnetic resonance, or transabdominal ultrasonographic images [3]. Adult patients (≥18 years old) who had not received initial treatment outside of the three hospitals were included in this study. The time from onset to hospital admission did not exceed 24 hours. Patients who were pregnant; had pancreatic cancer, liver cirrhosis, or coagulation system disease; and whose laboratory examinations were incomplete were excluded from this study.

All patients received standardized treatment in accordance with the guidelines for the management of AP [19]. The presence and persistence of OF were evaluated by the modified Marshall score during hospitalization.

### 2.2. Technical Protocols

Feature selection was applied to choose the features that had significant differences between the MOF group and non-MOF group. The combination of the training and validation sets for this study was obtained retrospectively. We used K-fold cross-validation for the training and validation set for internal validation, and it was also applied to build predictive models and obtain optimal features. To evaluate the predictive performance of our proposed models [20], we established a prospective cohort as a test set. The flow diagram of the training, validation, and test process of the prediction models is shown in Supplementary Figure 1. All authors had access to the study data and reviewed and approved the final manuscript. The study protocol was approved by the Research Ethics Commission of Daping Hospital (No.10,2018).

### 2.3. Data Collection

Demographic and clinical information and outcome data were extracted from electronic medical records. For the laboratory data, 23 features were chosen, including the complete blood count, coagulation profile, and serum biochemical tests. All data obtained on admission are shown in Supplementary Table 1.

### 2.4. Machine Learning

The models were based on machine learning algorithms with the inputting of variables that had significant differences (*p* < .05) in univariate analysis between AP patients with MOF or without MOF to predict the risk of MOF. Six machine learning algorithms were selected: support vector machine (SVM) algorithm, logistic regression analysis (LR), naive Bayes (NB) algorithm, quadratic discriminant analysis (QDA), adaptive boosting (AdaBoost), and back propagation network (BP); they were applied by using Matlab 2014. To select the optimal feature subset for each machine learning method, five-fold cross-validation was used for the training and validation set. Four of the five folds were used as the training set, and the remaining one was used as the validation set. Because each of the five folds was used as the validation set, the above process was repeated 20 times. Thereafter, a single optimal feature, optimal feature subset, and all features in corresponding models were generated.

### 2.5. Evaluation and Testing of the Machine Learning Models

The area under the curve (AUC) of the receiver operating characteristic (ROC) curve, sensitivity, and specificity were used to evaluate the predictive performance of the established models. These machine learning models trained on optimal feature subsets were then tested by a prospective cohort of 116 adult patients admitted to the three affiliated hospitals mentioned above.

### 2.6. Quantification of Feature Importance in the Optimal Feature Subset

We quantified the importance of each feature in the optimal feature subset in corresponding models by the method of stepwise elimination; we eliminated features one by one from the optimal feature subset (with replacements) to compare the AUC values of the remaining feature combinations. The importance of each feature was defined as:

(1) $$\begin{array}{c} \eta_{i}=\frac{\text{AUC\_optimal}-\text{AU}{\text{C}}_{i}}{\sum_{i=1}^{n}\left(\text{AU}{\text{C}}_{\text{optimal}}-\text{AU}{\text{C}}_{i}\right)} \end{array}$$

where *η*_*i*_ is the importance of the feature and *n* is the number of features in the optimal feature subset.

### 2.7. Statistical Analysis

Categorical variables were expressed as proportions. Continuous variables were expressed as median and interquartile range values. We compared the included variables by the Pearson chi-square test for categorical variables and the Student's *t*-test and nonparametric Mann–Whitney *U* test, respectively, for the continuous variables of the normal and skewed distribution. A two-sided *p* value of less than.05 was considered statistically significant. Analyses were performed with SPSS Statistics V.23.0.

## 3. Results

### 3.1. Demographic and Clinical Characteristics

A total of 447 patients with AP were included in this study. MOF occurred in 142 of the 447 patients (32%) in the whole cohort. Of these patients, 331 were retrospectively included in the training and validation set from July 2014 to May 2018 (101 with MOF and 230 without MOF). A total of 116 patients were prospectively selected as a test set from June 2018 to December 2019 (41 with MOF and 75 without MOF). Supplementary Table 2 shows the demographic and clinical characteristics of the 331 patients included in the training and validation set, and the clinical characteristics of patients included in the test set are summarized in Supplementary Table 3. Supplementary Table 4 lists the types and combinations of OF in different subsets of patients.

In the training and validation set, the median age of the patients was 48 years, ranging from 19 to 88 years, and 63% of the patients were male (Supplementary Table 2). Consistent with our previous reports [12], biliary tract disease (in 36% of patients) and hypertriglyceridemia (in 37% of patients) were the most common causes of AP. Of all of the patients, 175 were obese (body mass index [BMI] ≥25 kg/m^2^) (Supplementary Table 2). Statistically significant univariate features included the risk factors mentioned above, such as triglyceride levels, blood coagulability as measured by a coagulogram and thromboelastogram, and IL-6 levels. Patients with MOF had reduced platelet counts and high-density lipoprotein levels and elevated levels of alanine aminotransferase, aspartate aminotransferase, creatinine, and other substances. Interestingly, the white blood cell counts and calcium ion levels, which are the diagnostic criteria for the Systemic Inflammatory Response Syndrome (SIRS) score and elements of the Ranson score, were not significantly different between the MOF and non-MOF groups. No statistical differences were observed in gender, age, history of hypertension and diabetes, etiology, and BMI between the two groups (*p* > .05).

### 3.2. Predictive Performance of Machine Learning Models in the Validation Set

**Figure 1:**
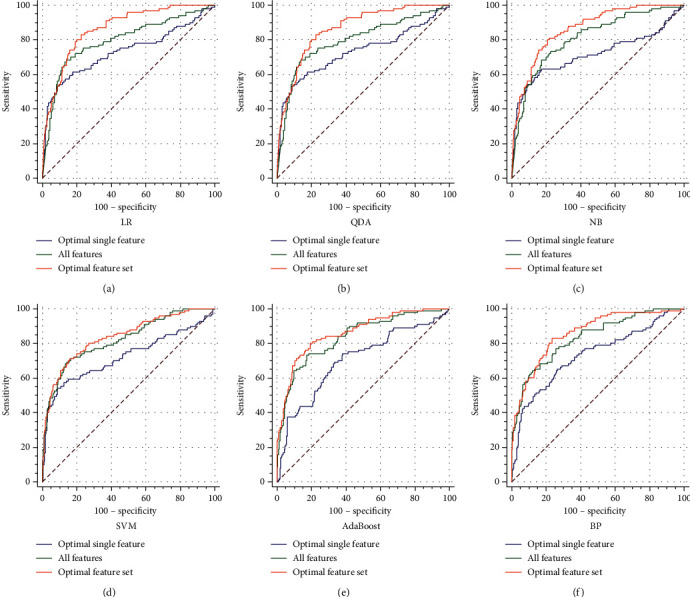
The ROC curves of different models in the validation set. (a) LR. (b) QDA. (c) NB. (d) SVM. (e) AdaBoost. (f) BP.

Eighteen features that had a significant difference between the two groups were introduced into the machine learning algorithms to determine which optimal feature subsets could effectively predict the risk of MOF in patients with AP (detailed in Supplementary Table 5). Creatinine was the optimal feature with the highest AUC values in all the candidate evaluations in the LR, QDA, NB, and SVM methods (0.7235 in LR, 0.7319 in QDA, 0.7153 in NB, and 0.7234 in SVM) (Supplementary Table 6). The kinetic time and blood urea nitrogen levels were the optimal features with the highest AUC values in all the candidate evaluations by AdaBoost and BP, respectively (0.7024 in the AdaBoost model; 0.7325 in the BP model) (Supplementary Table 6). Because different feature combinations had different predictive performances, the combinations with the maximum AUC values in the five-fold cross-validation were defined as the optimal feature subsets. Among these six models in the training and validation set, the QDA model obtained the highest AUC value (0.8653; 95% confidence interval [CI]: 0.824 to 0.900) in the subset of eight features including the levels of triglyceride and low-density lipoproteins (Table 1). The ROC curves obtained for the optimal feature subsets, the single features, and all of the features using K-fold cross-validation are shown in Figure 1. Table 1 shows the optimal feature subsets with the highest AUC values in each model.

Moreover, we compared the predictive performance obtained by the optimal feature subsets resulting from LR, QDA, NB, SVM, AdaBoost, and BP. The sensitivity (SEN), specificity (SPE), false-positive rate (FPR), false-negative rate (FNR), positive predictive value (PPV), negative predictive value (NPV), and accuracy of the six models are shown in Table 2. No significant differences were observed among these six models in PPV, NPV, accuracy, and AUC values (*p* > .05). The SEN of QDA and the SPE of LR were superior to the other models (*p* < .05) (Table 2).

### 3.3. Importance of each Feature in the Optimal Feature Subset of the Validation Set

**Figure 2:**
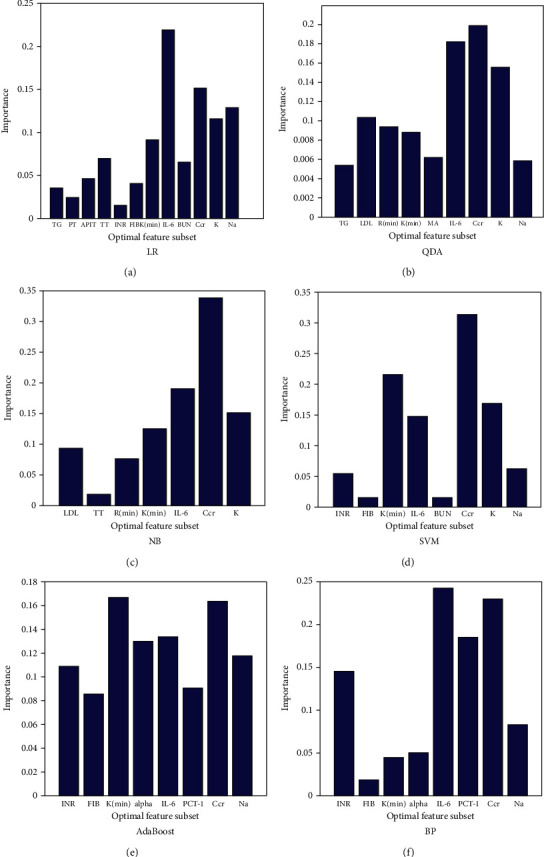
The importance of each feature in optimal feature subset in the validation set. (a) LR. (b) QDA. (c) NB. (d) SVM. (e) AdaBoost. (f) BP.

We quantified the importance of each feature in the optimal feature subset in corresponding models by the method of stepwise elimination. As is shown in Figure 2, the IL-6 level was the most important feature in both the LR and BP models. In the QDA, NB, and SVM models, the most predictive feature was the creatinine level. The kinetic time was the foremost feature in the AdaBoost model (Figure 2).

### 3.4. Predictive Performance of Machine Learning Models in the Test Set

**Figure 3:**
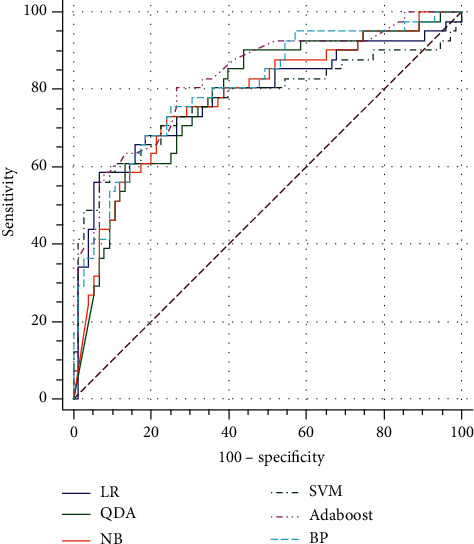
The ROC curves of optimal feature set of different models in test set.

To evaluate the predictive performance of each machine learning model trained by the optimal feature subsets, we performed an external evaluation and introduced a test set from a prospective cohort in the three hospitals. The AUC values obtained by the six models in the test set were 0.782 (95% CI: 0.694 to 0.853) for LR, 0.785 (95% CI: 0.686 to 0.848) for QDA, 0.779 (95% CI: 0.688 to 0.849) for NB, 0.772 (95% CI: 0.679 to 0.842) for SVM, 0.826 (95% CI: 0.740 to 0.888) for AdaBoost, and 0.805 (95% CI: 0.714 to 0.869) for BP (Table 3). The ROC curve obtained by each model in the test set is shown in Figure 3. No significant differences were observed among these four models regarding the SEN, FNR, PPV, NPV, accuracy, and AUC values (*p* > .05) (Table 3). The SPE and FPR of LR were best (*p* < .05). AdaBoost achieved the highest AUC value in the test set (Table 3).

### 3.5. Construction of Software for Predictive Models

To make use of this predictive tool in the hospital setting, we developed software based on machine learning. Clinicians can use this software easily by inputting the clinical parameters and laboratory results to train a predictive tool (Supplementary Figures 2 to 4). The first page provides the function of training and validation by using K-fold cross-validation to select the optimal feature subset. Six machine learning methods were employed in this software, and three manners of feature selection were provided (Supplementary Figure 2). Once the optimal feature subset was confirmed for a specific type of machine learning, the final predictive model was trained in the training and validation set and saved in a designated location. On the second page, one trained model is selected and its performance is evaluated in the test set (Supplementary Figure 3). On the third page, the primary data for admitted patients are input, and the verified predicting model, which was confirmed on the second page, is used to obtain a prediction probability for an upcoming patient (Supplementary Figure 4).

## 4. Discussion

MOF is the most important factor in determining the outcome of AP. Patients with predicted SAP benefit from being in the intensive care unit at an early phase of the disease [21, 22]. Single features such as age, comorbid conditions, and obesity might be important risk factors but are poor predictors for the development of MOF in these patients [19]. Here, we developed and validated predictive models for MOF complicated by MSAP and SAP to identify MOF at an early phase. Based on our previous research, we prospectively collected the test set, improved the generalization of models, validated the models by using an external test set, obtained a set of optimal features in each model, and quantified the importance of each feature.

The AdaBoost, QDA, and LR models were more likely to predict the risk of MOF complicated by AP. AdaBoost showed the best predictive performance in the test set. QDA was the most accurate model for predicting MOF with its highest AUC value and had superior SEN and NPVs in the training and validation set. The LR model had optimal SPE and PPVs in both the validation set and test set. The clinical risk factors of the included patients for MOF in this study were reported in previous studies [23–26]. Comorbidity, older age, obesity, and higher triglyceride levels were identified as independent risk factors for the development of OF in patients with AP. An etiology including hypertriglyceridemia, biliary disease, and alcoholism was not found to be an independent risk factor for OF, although patients with alcohol-induced AP may have a higher risk of an early onset of OF [27].

The optimal feature subsets of different machine learning methods could not be the same, but some variables were found in the optimal set of all machine learning methods, indicating that these features were critical for classification and for judging whether MOF would occur. The two representative features were IL-6 and creatinine levels. IL-6 was the foremost feature in the LR and BP models. Creatinine was the foremost feature in the QDA, NB, and SVM models. Kinetic time was the foremost feature in the AdaBoost model. Therefore, IL-6, creatinine, and the kinetic time played the most important roles in predicting the risk of MOF. Dambrauskas et al. performed a prospective study showing that IL-6 was one of the best indicators for diagnosing MSAP and SAP [9]. Another study demonstrated that higher serum levels of IL-6 were correlated with rates of OF and mortality [28]. Creatinine, an indicator of renal function, was found to contribute to the prediction of OF in SAP when its serum level was greater than or equal to 110 *μ*mol/L [29]. As part of the criteria for the severity stratification of OF in the modified Marshall score, levels of creatinine in patients with MOF were higher than those in patients without MOF in our study. The kinetic time, a parameter of a thromboelastogram reflecting the coagulation state, is equal to the generation time of thrombin [30]. The kinetic time in patients with MOF was prolonged compared with that in patients without MOF, suggesting a state of hypocoagulation. The relationship between inflammation reaction and coagulation dysfunction has been demonstrated [31–33]. Here, three features, creatinine, IL-6, and the kinetic time, were important independent variables for MOF, suggesting that these features should be monitored to prevent the occurrence of MOF in patients with AP.

With these models, it would be very convenient to get the predicted probability for MOF of patients with MSAP and SAP on admission; this timing is significantly superior to that for the evaluation of single features or intricate scoring systems such as APACHE II. Compared with conventional statistical methods, machine learning methods can detect complicated nonlinear relationships between various biochemical markers and a disease prognosis. The software we developed to train and test the predictive model can be conveniently used in daily clinical practice. Ensemble model can combine the models, and hopefully, this may improve the overall diagnostic accuracy. We will try to develop an ensemble model in our future work.

There were several limitations of our study. Firstly, the onset time of OF, which might be an aspect of the likely cause of OF and its outcome, was not included in this study. Secondly, our study reporting MOF as a binomial variable (present or absent) instead of at different stages of OF may lack the power to construct models that can predict the dynamic development of OF. Thirdly, Computerized tomography images are very important for evaluating the severity of AP but were not included in our study.

## 5. Conclusion

We developed effective models to predict the risk of MOF in patients with MSAP and SAP on admission. In the test set, AdaBoost was the superior predictive model, and IL-6 and creatinine levels were two representative predictive indicators.

## Figures and Tables

**Table 1:** The optimal feature subset of each machine learning method.

Features	TG	HDL	LDL	PT	APTT	TT	INR	FIB	R-time	K-time	*α*	MA	IL-6	PCT	BUN	Creatinine	K^+^	Na^+^	AUC
LR	√			√	√	√	√	√		√			√		√	√	√	√	0.8401
QDA	√		√						√			√	√			√	√	√	0.8653
NB			√			√			√	√			√			√	√		0.8646
SVM							√	√		√			√		√	√	√	√	0.8390
AdaBoost			√					√		√	√		√	√		√		√	0.8629
BP		√		√						√	√		√			√	√	√	0.8616

Abbreviations: TG: triglyceride; HDL: high-density lipoprotein; LDL: low-density lipoprotein; PT: prothrombin time; APTT: activated partial thromboplastin time; TT: thrombin time; INR: international normalized ratio; FIB: fibrinogen; R-time: reaction time; K-time: kinetic time; *α*: alpha angle; MA: maximum amplitude; IL-6: interleukin-6; PCT: procalcitonin; BUN: blood urea nitrogen; K^+^: potassium; Na^+^: sodium.

**Table 2:** Comparison of the predictive performance of different models in optimal feature subset in validation set.

Variable	LR (95% CI)	QDA (95% CI)	NB (95% CI)	SVM (95% CI)	AdaBoost (95% CI)	BP (95% CI)	*p* value
SEN	65.4% (55.2-74.5%)^a,c,d,e^	83.2% (75.7-90.6%)b	81.2% (73.4-88.9%)^b^	71.3% (62.3-80.3%)	80.2% (72.3-88.1%)^b^	83.2% (75.7-90.6%)^b^	**0.008**
SPE	90.0% (85.4-93.6%)	77.4% (71.5-82.4%)	78.3% (72.4-83.2%)	83.9% (78.6-88.3%)	80.4% (75.3-85.6%)	76.5% (71.0-82.0%)	**0.002**
FPR	10.0% (6.4-14.9%)	22.6% (17.6-28.5%)	21.7% (16.8-27.6%)	16.1% (11.7-21.4%)	19.57% (14.4-24.7%)	23.5% (18.0-29.0%)	0.002
FNR	35.6% (25.5-46.8%)^a,c,d,e^	16.8% (9.4-24.3%)^b^	9.1% (11.1-26.6%)^b^	28.7% (10.7-37.7%)	19.8% (11.9-27.7%)^b^	26.8% (9.4-24.3%)^b^	0.008
PPV	73.3% (64.0-82.6%)	61.3% (53.1-69.6%)^a^	61.7% (53.3-70.0%)^b,c^	65.5% (56.4-74.5%)^a^	64.3% (55.8-72.8%)	60.9% (52.6-69.1%)	0.437
NPV	85.5% (81.0-90.0%)	91.2% (87.2-95.3%)	90.4% (86.3-94.5%)	86.9% (82.4-91.4%)	90.2% (86.1-94.3%)	91.2% (87.2-95.2%)	0.239
Accuracy	82.2% (78.0-86.3%)	78.9% (74.4-83.3%)	78.9% (74.4-83.3%)	79.8% (75.4-81.4%)	80.4% (76.1-84.7%)	78.5% (74.1-83.0%)	0.862
AUC	0.840 (0.796-0.878)	0.865 (0.824-0.900)	0.864 (0.823-0.899)	0.839 (0.795-0.877)	0.863 (0.821-0.898)	0.862 (0.820-0.897)	/

^a^Compared with QDA, *p* < 0.05; ^b^Compared with LR, *p* < 0.05; ^c^Compared with NB, *p* < 0.05; ^d^Compared with AdaBoost, *p* < 0.05; ^e^Compared with BP, *p* < 0.05. *p* value denoted the overall statistical result for the four models.

**Table 3:** Comparison of the predictive performance of different models in optimal feature subset in test set.

Variable	LR (95%CI)	QDA (95%CI)	NB (95%CI)	SVM (95%CI)	Adaboost (95%CI)	BP (95%CI)	*p* value
SEN	58.54% (42.20%-73.30%)	60.98% (44.54%-75.38%)	73.17% (56.69%-85.25%)	60.98% (44.54%-75.38%)	80.49% (64.63%-90.63%)	75.61% (59.36%-87.09%)	0.15
SPE	93.33% (84.47%-95.52%)^a,c,d,e^	86.67% (76.39%-93.08%)^d^	76.00% (64.50%-84.79%)^b^	89.33% (79.54%-94.95%)^d^	73.33% (61.66%-82.58%)^a,b,f^	74.67% (63.08%-83.69%)^a,b^	**0.001**
FPR	6.67% (1.02%-12.32%)^a,c,d,e^	13.33% (5.64%-21.03%) ^d^	24.00% (14.33%-33.67%) ^b^	10.67% (3.68%-17.66%) ^d^	26.67% (16.66%-36.67%) ^a,b,f^	25.33% (15.49%-35.17%) ^a,b^	**0.001**
FNR	41.46% (26.38%-56.54%)	39.02% (24.09%-77.80%)	26.83% (13.27%-40.39%)	39.02% (24.09%-77.80%)	19.51% (7.38%-31.64%)	24.39% (11.25%-37.53%)	0.15
PPV	82.76% (63.51%-93.47%)	71.43% (53.48%-84.76%)	62.50% (47.33%-75.68%)	75.76% (57.37%-88.26%)	62.26% (47.87%-74.88%)	62.00% (47.16%-75.00%)	0.281
NPV	93.33% (84.47%-97.52%)	80.25% (69.61%-87.95%)	83.82% (72.47%-91.27%)	80.72% (70.29%-88.25%)	87.30% (75.96%-93.97%)	84.85% (73.44%-92.11%)	0.87
Accuracy	80.3% (73.0-87.7%)	78.5% (71.1-85.9%)	75.0% (67.0-83.0%)	79.3% (71.8-86.8%)	75.9% (68.0-83.8%)	75.0% (67.0-83.0%)	0.831
AUC	0.782 (0.694-0.853)	0.785 (0.686-0.848)	0.779 (0.688-0.849)	0.772 (0.679-0.842)	0.826 (0.740-0.888)	0.805 (0.714-0.869)	/

^a^Compared with QDA, *p* < 0.05; ^b^Compared with LR, *p* < 0.05; ^c^Compared with NB, *p* < 0.05; ^d^Compared with AdaBoost, *p* < 0.05; ^e^Compared with BP, *p* < 0.05; ^f^Compared with SVM, *p* < 0.05. *p* value denoted the overall statistical result for the four models.

## Data Availability

The datasets used and analyzed during the current study are available from the corresponding author on reasonable request.

## Abbreviations

AP: Acute pancreatitis
OF: Organ failure
SAP: Severe acute pancreatitis
MOF: Multiple organ failure
APACHE II: Acute physiology and chronic health evaluation II;
IL: Interleukin
SVM: Support vector machine
LR: Logistic regression analysis
NB: Naive Bayes
QDA: Quadratic discriminant analysis
AdaBoost: Adaptive boosting
BP: Back propagation network
AUC: Area under the curve
ROC: Receiver operating characteristic
BMI: Body mass index
SIRS: Systemic inflammatory response syndrome
SEN: Sensitivity
SPE: Specificity
FPR: False-positive rate
FNR: False-negative rate
PPV: Positive predictive value
NPV: Negative predictive value.

## Supplementary Materials

Supplementary Materials Supplementary Figure 1: the flow diagram of the training, validation, and test of the prediction models. Supplementary Figure 2: the first page of the software. The first page provides the function of training and validation by using K-fold cross-validation to select the optimal feature subset. Supplementary Figure 3: the second page of the software. On the second page, one trained model is selected and its performance is evaluated in the test set. Supplementary Figure 4: the third page of the software. The primary data for admitted patients are input, and the verified predicting model, which was confirmed on the second page, is used to obtain a prediction probability for an upcoming patient. Supplementary Table 1: laboratory data obtained on admission of all patients. Supplementary Table 2: demographics and clinical characteristics of patients in the training and validation set. Supplementary Table 3: demographics and clinical characteristics of patients in test set. Supplementary Table 4: type and combination of organ failure in different sets of patients. Supplementary Table 5: the input features for feature selection by using K-fold cross validation. Supplementary Table 6: the predictive performance by single optimal feature in all candidate feature subset of six models.
