# Supplementary material for: Prediction of Multiple Organ Failure Complicated by Moderately Severe or Severe Acute Pancreatitis Based on Machine Learning: A Multicenter Cohort Study
Source: Mediators Inflamm. 2021 May 3;2021:5525118. doi: 10.1155/2021/5525118 (PMC8112913; doi:10.1155/2021/5525118)
Supplement: Supplementary Materials — Supplementary Figure 1: the flow diagram of the training, validation, and test of the prediction models. Supplementary Figure 2: the first page of the software. The first page provides the function of training and validation by using K-fold cross-validation to select the optimal feature subset. Supplementary Figure 3: the second page of the software. On the second page, one trained model is selected and its performance is evaluated in the test set. Supplementary Figure 4: the third page of the software. The primary data for admitted patients are input, and the verified predicting model, which was confirmed on the second page, is used to obtain a prediction probability for an upcoming patient. Supplementary Table 1: laboratory data obtained on admission of all patients. Supplementary Table 2: demographics and clinical characteristics of patients in the training and validation set. Supplementary Table 3: demographics and clinical characteristics of patients in test set. Supplementary Table 4: type and combination of organ failure in different sets of patients. Supplementary Table 5: the input features for feature selection by using K-fold cross validation. Supplementary Table 6: the predictive performance by single optimal feature in all candidate feature subset of six models. [file 5525118.f1.zip › 5525118.f2.docx]

| **Supplementary Table 2. Demographics and clinical characteristics of patients in the training and validation set.** | | | | | | |  |
| --- | --- | --- | --- | --- | --- | --- | --- |
| Demographics and clinical characteristics | | | All Patients (n=331) | MOF n=101 (31%) | Non-MOF n=230 (69%) | *p* value |  |
| Median age, years, (IQR) | | | 48.00(39.00-59.00) | 49.00(40.00-63.00) | 47.00(39.00-58.00) | 0.271 |  |
| Male sex, N (%) | |  | 210(63) | 60(60) | 150(65) | 0.312 |  |
| Etiology, N (%) | | |  |  |  | 0.595 |  |
| Hypertriglyceridemia | |  | 124(37) | 38(38) | 86(37) |  |  |
| Biliary | |  | 118(36) | 36(36) | 82(36) |  |  |
| Alcoholic | |  | 29(9) | 6(6) | 23(10) |  |  |
| Other | |  | 60(18) | 21(21) | 39(17) |  |  |
| BMI, kg/m2 | |  | 25.53±3.79 | 25.61±3.09 | 25.49±4.05 | 0.788 |  |
| Obese (BMI>=25), N (%) | | | 175(55) | 51(50) | 124(54) | 0.972 |  |
| History of hypertension, N (%) | | | 70(21) | 27(27) | 43(19) | 0.099 |  |
| History of diabetes, N (%) | | | 41(12） | 13(13) | 28(12) | 0.859 |  |
| **Complete blood cell count** | | | | | | |  |
| White cell count, (*10~9/L) | | | 13.80(9.49-17.56) | 14.57(11.52-9.55) | 13.64(9.49-11.35) | 0.262 |  |
| Neutrophil count, (*10~9/L) | | | 11.58(8.16-15.65) | 11.47(9.11-15.76) | 11.71(7.92-15.68) | 0.351 |  |
| Hematocrit, % | |  | 37.60(29.90-45.00) | 34.10(26.10-46.65) | 37.95(32.53-44.40) | 0.161 |  |
| Platelets count, (*10~9/L) | | | 173.00(119.00-239.00) | 148.00(94.50-249.50) | 174.50(128.00-236.00) | 0.04 |  |
| mean platelet volume, fL | | | 12.10(10.90-13.50) | 12.10(11.00-13.70) | 12.00(10.90-13.40) | 0.988 |  |
| platelet distribution width, % | | | 16.60(14.10-18.63) | 16.70(15.00-18.30) | 16.50(13.40-19.38) | 0.402 |  |
| platelet-large cell ratio, % | | | 37.80(27.60-47.20) | 40.90(32.30-49.20) | 36.35(25.07-46.27) | 0.008 |  |
| plateletocrit, % | | | 0.22(0.15-0.30) | 0.21(0.13-0.29) | 0.22(0.17-0.29) | 0.279 |  |
| **Liver function** | | | | | | |  |
| ALT, (IU/L) | |  | 27.10(17.20-52.70) | 38.70(20.65-107.80) | 25.40(16.00-43.87) | 0.000 |  |
| AST, (IU/L) | |  | 37.00(23.50-66.40) | 61.10(33.15-134.10) | 30.90(22.35-49.30) | 0.000 |  |
| GGT, (IU/L) | |  | 72.00(34.65-162.38) | 79.60(37.33-189.03) | 70.80(31.35-143.55) | 0.131 |  |
| ALP, (IU/L) | |  | 87.75(66.35-127.95) | 95.90(62.50-150.45) | 86.00(68.40-119.90) | 0.432 |  |
| Triglyceride, (mmol/L) | |  | 2.80(1.32-6.02) | 2. 80(1.47-6.42) | 2.77(1.27-5.80) | 0.416 |  |
| Total cholesterol, (mmol/L) | | | 4.01(2.80-5.75) | 3.45(2.06-5.31) | 4.22(2.94-5.76) | 0.008 |  |
| high-density lipoprotein, (mmol/L) | | | 0.77(0.54-1.05) | 0.62(0.42-0.87) | 0.80(0.60-1.11) | 0.000 |  |
| low density lipoprotein, (mmol/L) | | | 2.07(1.29-2.95) | 1.51(0.85-2.67) | 2.19(1.46-3.01) | 0.001 |  |
| **Renal function** | | | | | | |  |
| BUN, (mmol/L) | |  | 5.71(3.98-9.30) | 9.34(5.75-14.79) | 5.26(3.70-7.14) | 0.000 |  |
| Creatinine, (umol/L) | |  | 68.70(52.70-110.10) | 124.60(64.10-252.00) | 63.55(50.95-84.07) | 0.000 |  |
| **Biochemical indexes** | | | | | | |  |
| K+, (mmol/L) | |  | 3.92(3.51-4.35) | 4.20(3.70-4.66) | 3.84(3.47-4.21) | 0.000 |  |
| Na+ (mmol/L) | |  | 136.50(133.40-137.50) | 138.10(135.60-142.15) | 135.40(132.77-138.53) | 0.000 |  |
| Calcium, (mmol/L) | |  | 1.98(1.79-2.16) | 1.96(1.74-2.16) | 1.99(1.81-2.16) | 0.581 |  |
| **Pancreatic enzyme** | | | | | | |  |
| Amylopsin, (IU/L) | |  | 227.80(75.52-746.75) | 344.50(104.15-996.00) | 195.95(65.02-622.72) | 0.022 |  |
| Lipase, (IU/L) | | | 189.00(62.83-567.60) | 262.75(71.55-816.80) | 167.85(59.72-430.50) | 0.022 |  |
| **Inflammatory markers** | | | | | | |  |
| C-reactive protein, (mg/L) | | | 144.90(52.80-200.00) | 174.20(93.22-204.50) | 130.00(37.70-200.00) | 0.042 |  |
| Interleukin-6, (pg/ml) | |  | 61.06(14.15-158.60) | 105.25(43.99-362.47) | 42.30(7.40-113.80) | 0.000 |  |
| procalcitonin, (ng/ml) | |  | 0.95(0.37-3.96) | 3.74(0.65-12.98) | 0.70(0.23-1.79) | 0.000 |  |
| **Coagulogram** | | |  |  |  |  |  |
| PT, (s) | |  | 12.80(11.90-14.50) | 14.10(12.30-15.80) | 12.65(11.70-13.80) | 0.000 |  |
| APTT, (s) | |  | 31.20(27.80-37.4) | 36.10(29.25-45.55) | 30.30(27.20-33.62) | 0.000 |  |
| TT, (s) | |  | 15.50(14.10-18.80) | 16.60(14.95-20.30) | 15.00(13.60-17.42) | 0.000 |  |
| Fibrinogen, (g/L) | |  | 4.45(3.30-5.81) | 3.84(2.92-4.93) | 4.64(3.68-5.99) | 0.000 |  |
| INR | |  | 1.11(1.04-1.25) | 1.20(1.05-1.39) | 1.10(1.03-1.20) | 0.000 |  |
| D-Dimer, (mg/L) | |  | 2.70(0.99-4.77) | 3.08(1.37-5.87) | 2.43(0.73-4.72) | 0.014 |  |
| **Thrombelastogram** | | |  |  |  |  |  |
| R-time, minutes | |  | 5.80(4.70-7.00) | 6.40(5.70-4.70) | 5.70(4.70-6.60) | 0.001 |  |
| K-time, minutes | |  | 1.50(1.20-1.90) | 1.90(1.30-2.90) | 1.40(1.20-1.70) | 0.000 |  |
| α, degrees | |  | 69.40(62.30-73.00) | 64.20(52.70-71.65) | 70.30(65.05-73.20) | 0.000 |  |
| MA, mm | |  | 68.20(61.80-72.70) | 64.20(55.00-70.25) | 69.35(64.30-73.50) | 0.000 |  |
| Ly30, % | |  | 1.02(0.48-1.57) | 0.65(0.26-1.03) | 1.19(0.42-1.95) | 0.211 |  |
| P values were calculated by t test, Mann-Whitney U test, χ^2^ test, or Fisher’s exact test, as appropriate. Abbreviations: ALT: alanine aminotransferase, AST: aspartate aminotransferase, GGT: gamma-glutamyl transpeptidase, ALP: alkaline phosphatase, BUN: blood urea nitrogen, K+: potassium, Na+: sodium, PT: prothrombin time, APTT: activated partial thromboplastin time, INR: international normalized ratio. R-time: Reaction time, K-time: Kinetic time, α: Alpha angle, MA: Maximum amplitude. | | | | | | |  |
|  |  |  |  |  |  |  |  |
|  |  |  |  |  |  |  |  |
|  |  |  |  |  |  |  |  |
|  |  |  |  |  |  |  |  |
|  |  |  |  |  |  |  |  |
