# Supplementary material for: Prediction of Multiple Organ Failure Complicated by Moderately Severe or Severe Acute Pancreatitis Based on Machine Learning: A Multicenter Cohort Study
Source: Mediators Inflamm. 2021 May 3;2021:5525118. doi: 10.1155/2021/5525118 (PMC8112913; doi:10.1155/2021/5525118)
Supplement: Supplementary Materials — Supplementary Figure 1: the flow diagram of the training, validation, and test of the prediction models. Supplementary Figure 2: the first page of the software. The first page provides the function of training and validation by using K-fold cross-validation to select the optimal feature subset. Supplementary Figure 3: the second page of the software. On the second page, one trained model is selected and its performance is evaluated in the test set. Supplementary Figure 4: the third page of the software. The primary data for admitted patients are input, and the verified predicting model, which was confirmed on the second page, is used to obtain a prediction probability for an upcoming patient. Supplementary Table 1: laboratory data obtained on admission of all patients. Supplementary Table 2: demographics and clinical characteristics of patients in the training and validation set. Supplementary Table 3: demographics and clinical characteristics of patients in test set. Supplementary Table 4: type and combination of organ failure in different sets of patients. Supplementary Table 5: the input features for feature selection by using K-fold cross validation. Supplementary Table 6: the predictive performance by single optimal feature in all candidate feature subset of six models. [file 5525118.f1.zip › 5525118.f3.docx]

| **Supplementary table 3. Demographics and clinical characteristics of patients in test set.** | | | | | | |
| --- | --- | --- | --- | --- | --- | --- |
| Demographics and clinical characteristics | | | All Patients (n=116) | MOF n=41 (35%) | Non-MOF n=75 (65%) | *p* value |
| Median age, years, (IQR) | | | 49.74±15.54 | 52.58±15.64 | 48.19±15.37 | 0.146 |
| Male sex, N (%) | |  | 71(61) | 27(66) | 44(59） | 0.313 |
| Etiology, N (%) | | |  |  |  | 0.468 |
| Hypertriglyceridemia | |  | 49(37) | 17(41) | 32(43) |  |
| Biliary | |  | 33(28) | 11(27) | 22(29) |  |
| Alcoholic | |  | 7(6) | 6(15) | 1(1) |  |
| Other | |  | 27(23) | 12(29) | 15(20) |  |
| BMI, kg/m2 | |  | 25.53±3.79 | 25.42±3.61 | 25.53±3.79 | 0.898 |
| Obese (BMI>=25), N (%) | | | 47(41) | 14(34) | 33(44) | 0.301 |
| History of hypertension, N (%) | | | 28(24) | 16(39) | 12(16) | 0.006 |
| History of diabetes, N (%) | | | 19(16） | 3(7) | 16(21) | 0.067 |
| **Complete blood cell count** | | | | | | |
| White cell count, (*10~9/L) | | | 12.88(10.30-17.45） | 12.91(9.83-17.05) | 12.88(10.43-17.69) | 0.749 |
| Neutrophil count, (*10~9/L) | | | 11.45(8.50-15.62) | 11.26(7.95-14.95) | 11.85(8.97-15.69) | 0.642 |
| Hematocrit, % | |  | 36.65±9.64 | 34.78±11.11 | 37.68±8.65 | 0.123 |
| Platelets count, (*10~9/L) | | | 183.50(125.75-245.25) | 167.00(113.50-228.50) | 188.00(128.50-252.00) | 0.202 |
| mean platelet volume, fL | | | 12.10(10.90-13.07) | 12.40(11.40-13.35) | 12.00(10.75-12.80) | 0.058 |
| platelet distribution width, % | | | 16.50(14.97-17.30) | 16.90(15.35-18.90) | 16.40(14.60-17.00) | 0.012 |
| platelet-large cell ratio, % | | | 41.40(30.27-46.95) | 40.70(30.05-47.40) | 41.70(30.40-46.85) | 0.827 |
| plateletocrit, % | | | 0.23(0.17-0.29) | 0.21(0.16-0.28) | 0.24(0.19-0.29) | 0.229 |
| **Liver function** | | | | | | |
| ALT, (IU/L) | |  | 35.05(21.92-59.62) | 37.90(23.20-93.60) | 30.00(20.80-53.90) | 0.279 |
| AST, (IU/L) | |  | 44.40(27.85-90.60) | 68.10(37.40-118.20) | 35.80(25.50-73.20) | 0.009 |
| GGT, (IU/L) | |  | 99.70(41.07-191.75) | 113.20(41.40-171.00) | 90.00(37.20-224.00) | 0.805 |
| ALP, (IU/L) | |  | 94.80(71.70-130.67) | 88.10(67.10-123.70) | 99.00(73.80-156.20) | 0.043 |
| Triglyceride, (mmol/L) | |  | 2.64(1.37-9.86) | 2.86(1.31-5.53) | 2.63(1.38-10.63) | 0.842 |
| Total cholesterol, (mmol/L) | | | 4.15(2.72-7.03) | 3.74(2.22-4.91) | 4.32(2.79-8.32) | 0.067 |
| high-density lipoprotein, (mmol/L) | | | 0.69(0.50-1.06) | 0.64(0. 49-0.85) | 0.78(0.52-1.14) | 0.146 |
| low density lipoprotein, (mmol/L) | | | 1.93(1.12-2.77) | 1.72(1.09-2.60) | 2.02(1.23-2.84) | 0.407 |
| **Renal function** | | | | | | |
| BUN, (mmol/L) | |  | 5.67(4.42-8.79) | 9.77(4.97-15.81) | 5.04(4.11-6.67) | 0.000 |
| Creatinine, (umol/L) | |  | 69.65(50.82-102.00) | 101.70(53.80-251.70) | 64.00(50.10-82.20) | 0.001 |
| **Biochemical indexes** | | | | | | |
| K^+^, (mmol/L) | |  | 4.06(3.62-4.53) | 4.16(3.85-4.66) | 3.99(3.53-4.39) | 0.049 |
| Na^+^ (mmol/L) | |  | 136.00(132.65-138.50) | 137.10(133.05-139.50) | 135.70(132.00-138.50) | 0.215 |
| Calcium, (mmol/L) | |  | 2.03(1.79-2.21) | 1.94(1.69-2.19) | 2.05(1.84-2.23) | 0.162 |
| **Pancreatic enzyme** | | | | | | |
| Amylopsin, (IU/L) | |  | 298.60(108.00-973.10) | 468.65(170.05-1074.95) | 214.35(97.40-852.28) | 0.059 |
| Lipase, (IU/L) | | | 332.20(94.65-680.33) | 469.78(165.50-788.00) | 235.50(87.73-630.58) | 0.148 |
| **Inflammatory markers** | | | | | | |
| C-reactive protein, (mg/L) | | | 143.05(61.40-200.00) | 164.80(99.12-212.45) | 126.95(35.30-200.00) | 0.268 |
| Interleukin-6, (pg/ml) | |  | 72.25(21.18-72.25) | 149.70(50.44-413.38) | 46.76(15.66-126.88) | 0.000 |
| procalcitonin, (ng/ml) | |  | 1.33(0.36-6.41) | 5.15(1.22-12.07) | 0.67(0.24-2.16) | 0.000 |
| **Coagulogram** | | |  |  |  |  |
| PT, (s) | |  | 12.80(11.70-14.30) | 13.60(12.32-15.62) | 12.60(11.50-13.80) | 0.009 |
| APTT, (s) | |  | 30.00(27.63-33.60) | 30.85(28.40-40.80) | 29.40(27.40-32.40) | 0.015 |
| TT, (s) | |  | 15.40(13.60-18.60) | 16.60(13.85-21.12) | 14.30(13.40-17.40) | 0.011 |
| Fibrinogen, (g/L) | |  | 4.57(3.21-6.89) | 4.21(2.85-6.30) | 4.78(3.30-6.97) | 0.303 |
| INR | |  | 1.12(1.02-1.26) | 1.18(1.08-1.36) | 1.11(1.01-1.22) | 0.023 |
| D-Dimer, (mg/L) | |  | 1.74(0.54-3.44) | 2.09(0.82-6.14) | 1.07(0.49-3.13) | 0.087 |
| **Thrombelastogram** | | |  |  |  |  |
| R-time, minutes | |  | 5.55(4.80-6.67) | 6.20(5.20-8.70) | 5.30(4.50-6.12) | 0.001 |
| K-time, minutes | |  | 1.40(1.20-1.87) | 1.60(1.20-2.30) | 1.35(1.20-1.80) | 0.029 |
| α, degrees | |  | 69.60(64.22-72.90) | 67.40(58.80-71.70) | 70.10(64.80-72.97) | 0.121 |
| MA, mm | |  | 67.10(61.63-71.35) | 65.10(58.80-70.80) | 68.30(63.42-71.40) | 0.225 |
| Ly30, % | |  | 2.95(0.76-5.14) | 3.83(0.61-8.27) | 2.50(0.02-4.99) | 0.991 |
| P values were calculated by t test, Mann-Whitney U test, χ^2^ test, or Fisher’s exact test, as appropriate. Abbreviations: ALT: alanine aminotransferase, AST: aspartate aminotransferase, GGT: gamma-glutamyl transpeptidase, ALP: alkaline phosphatase, BUN: blood urea nitrogen, K^+^: potassium, Na^+^: sodium, PT: prothrombin time, APTT: activated partial thromboplastin time, INR: international normalized ratio. R-time: Reaction time, K-time: Kinetic time, α: Alpha angle, MA: Maximum amplitude | | | | | | |
|  |  |  |  |  |  |  |
|  |  |  |  |  |  |  |
|  |  |  |  |  |  |  |
|  |  |  |  |  |  |  |
|  |  |  |  |  |  |  |
