# Supplementary material for: Prediction of Multiple Organ Failure Complicated by Moderately Severe or Severe Acute Pancreatitis Based on Machine Learning: A Multicenter Cohort Study
Source: Mediators Inflamm. 2021 May 3;2021:5525118. doi: 10.1155/2021/5525118 (PMC8112913; doi:10.1155/2021/5525118)
Supplement: Supplementary Materials — Supplementary Figure 1: the flow diagram of the training, validation, and test of the prediction models. Supplementary Figure 2: the first page of the software. The first page provides the function of training and validation by using K-fold cross-validation to select the optimal feature subset. Supplementary Figure 3: the second page of the software. On the second page, one trained model is selected and its performance is evaluated in the test set. Supplementary Figure 4: the third page of the software. The primary data for admitted patients are input, and the verified predicting model, which was confirmed on the second page, is used to obtain a prediction probability for an upcoming patient. Supplementary Table 1: laboratory data obtained on admission of all patients. Supplementary Table 2: demographics and clinical characteristics of patients in the training and validation set. Supplementary Table 3: demographics and clinical characteristics of patients in test set. Supplementary Table 4: type and combination of organ failure in different sets of patients. Supplementary Table 5: the input features for feature selection by using K-fold cross validation. Supplementary Table 6: the predictive performance by single optimal feature in all candidate feature subset of six models. [file 5525118.f1.zip › 5525118.f4.docx]

| **Supplementary table 4. Type and combination of organ failure in different sets of patients** | | | | | | | |
| --- | --- | --- | --- | --- | --- | --- | --- |
|  |  |  | Training and validation set | | Test set | | *p* value |
| Multiple organ failure (any two or more organ systems) | | | 101/331 (31) | | 41/116 (35) | | 0.336 |
|  |  |  |  |  |  |  |  |
| Any two organ systems | | |  |  |  |  |  |
| Cardiovascular and respiratory | | | 48/101(48) | | 6/41(15) | | 0.000 |
| Respiratory and renal | | | 25/101(25) | | 23/41(56) | | 0.001 |
| Renal and cardiovascular | | | 3/101(3) | | 4/41(10) | | 0.106 |
| All three organ systems | | | 25/101(25) | | 8/41() | | 0.503 |
| All values in parentheses are percentages. Data represent the longest persistent episode of organ failure per system. In case episodes of different organ systems were equal in duration, the organ system involved in the first episode was used. P values were calculated by χ^2^ test or Fisher’s exact test. | | | | | | | |
|  |  |  |  |  |  |  |  |
|  |  |  |  |  |  |  |  |
|  |  |  |  |  |  |  |  |
|  |  |  |  |  |  |  |  |
