# Supplementary material for: Prediction of Multiple Organ Failure Complicated by Moderately Severe or Severe Acute Pancreatitis Based on Machine Learning: A Multicenter Cohort Study
Source: Mediators Inflamm. 2021 May 3;2021:5525118. doi: 10.1155/2021/5525118 (PMC8112913; doi:10.1155/2021/5525118)
Supplement: Supplementary Materials — Supplementary Figure 1: the flow diagram of the training, validation, and test of the prediction models. Supplementary Figure 2: the first page of the software. The first page provides the function of training and validation by using K-fold cross-validation to select the optimal feature subset. Supplementary Figure 3: the second page of the software. On the second page, one trained model is selected and its performance is evaluated in the test set. Supplementary Figure 4: the third page of the software. The primary data for admitted patients are input, and the verified predicting model, which was confirmed on the second page, is used to obtain a prediction probability for an upcoming patient. Supplementary Table 1: laboratory data obtained on admission of all patients. Supplementary Table 2: demographics and clinical characteristics of patients in the training and validation set. Supplementary Table 3: demographics and clinical characteristics of patients in test set. Supplementary Table 4: type and combination of organ failure in different sets of patients. Supplementary Table 5: the input features for feature selection by using K-fold cross validation. Supplementary Table 6: the predictive performance by single optimal feature in all candidate feature subset of six models. [file 5525118.f1.zip › 5525118.f5.docx]

| Supplementary table 5. The input features for feature selection by using K-fold cross validation | | | | | | | | | |
| --- | --- | --- | --- | --- | --- | --- | --- | --- | --- |
| **Variables** | | |  | | |  | | |  |
| **Laboratory findings** | | |  |  |  |  |  |  |  |
| **Liver function** | |  |  |  |  |  |  |  |  |
| triglyceride (TG) |  |  |  | high-density lipoprotein (HDL) | |  |  | low density lipoprotein (LDL) | |
| **Coagulogram** | |  |  |  |  |  |  |  |  |
| prothrombin time (PT) | activated partial thromboplastin time (APTT) | | | Fibrinogen (FIB) | | thrombin time (TT) | | international normalized ratio (INR) | |
| **Thrombelastogram (TEG)** | |  |  |  |  |  |  |  |  |
| Reaction time (R-time) | | Kinetic time (K-time) | | Alpha angle (α angle) | |  |  | Maximum amplitude (MA) | |
| **Inflammatory markers** | |  |  |  |  |  |  |  |  |
| Interleukin-6 (IL-6) | |  |  | plateletocrit (PCT) | |  |  |  |  |
| **Renal function** | | |  |  |  |  |  |  |  |
| blood urea nitrogen (BUN) | |  |  | creatinine (Cr) | |  |  |  |  |
| **Biochemical indexes** | |  |  |  |  |  |  |  |  |
| potassium ion (K^+^) | |  |  | Calcium ion (Ca^2+^) | |  |  | sodium ion (Na^+^) | |
|  |  |  |  |  |  |  |  |  |  |
|  |  |  |  |  |  |  |  |  |  |
|  |  |  |  |  |  |  |  |  |  |
|  |  |  |  |  |  |  |  |  |  |
