# Supplementary material for: Prediction of Multiple Organ Failure Complicated by Moderately Severe or Severe Acute Pancreatitis Based on Machine Learning: A Multicenter Cohort Study
Source: Mediators Inflamm. 2021 May 3;2021:5525118. doi: 10.1155/2021/5525118 (PMC8112913; doi:10.1155/2021/5525118)
Supplement: Supplementary Materials — Supplementary Figure 1: the flow diagram of the training, validation, and test of the prediction models. Supplementary Figure 2: the first page of the software. The first page provides the function of training and validation by using K-fold cross-validation to select the optimal feature subset. Supplementary Figure 3: the second page of the software. On the second page, one trained model is selected and its performance is evaluated in the test set. Supplementary Figure 4: the third page of the software. The primary data for admitted patients are input, and the verified predicting model, which was confirmed on the second page, is used to obtain a prediction probability for an upcoming patient. Supplementary Table 1: laboratory data obtained on admission of all patients. Supplementary Table 2: demographics and clinical characteristics of patients in the training and validation set. Supplementary Table 3: demographics and clinical characteristics of patients in test set. Supplementary Table 4: type and combination of organ failure in different sets of patients. Supplementary Table 5: the input features for feature selection by using K-fold cross validation. Supplementary Table 6: the predictive performance by single optimal feature in all candidate feature subset of six models. [file 5525118.f1.zip › 5525118.f6.docx]

| Supplementary table 6. The predictive performance by single optimal feature in all candidate feature subset of six models. | | | | | | | | | | |
| --- | --- | --- | --- | --- | --- | --- | --- | --- | --- | --- |
| No. | Models | single optimal feature | | AUC | Sn | Sp |  |  |  |  |
| 1 | LR | Creatinine | | 0.7235 | 54.46% | 90.87% |  |  |  |  |
| 2 | QDA | Creatinine | | 0.7319 | 53.47% | 91.30% |  |  |  |  |
| 3 | NB | Creatinine | | 0.7153 | 63.37% | 82.61% |  |  |  |  |
| 4 | SVM | Creatinine | | 0.7234 | 54.46% | 91.30% |  |  |  |  |
| 5 | AdaBoost | K-time | | 0.7024 | 74.26% | 61.74% |  |  |  |  |
| 6 | BP | BUN | | 0.7325 | 65.35% | 73.48% |  |  |  |  |
| Abbreviations: K-time: Kinetic time, BUN: blood urea nitrogen, Sn: sensitivity, Sp: specificity. | | | | | | |  |  |  |  |
|  |  |  |  |  |  |  |  |  |  |  |
|  |  |  |  |  |  |  |  |  |  |  |
