# Supplementary material for: Prediction of Multiple Organ Failure Complicated by Moderately Severe or Severe Acute Pancreatitis Based on Machine Learning: A Multicenter Cohort Study
Source: Mediators Inflamm. 2021 May 3;2021:5525118. doi: 10.1155/2021/5525118 (PMC8112913; doi:10.1155/2021/5525118)
Supplement: Supplementary Materials — Supplementary Figure 1: the flow diagram of the training, validation, and test of the prediction models. Supplementary Figure 2: the first page of the software. The first page provides the function of training and validation by using K-fold cross-validation to select the optimal feature subset. Supplementary Figure 3: the second page of the software. On the second page, one trained model is selected and its performance is evaluated in the test set. Supplementary Figure 4: the third page of the software. The primary data for admitted patients are input, and the verified predicting model, which was confirmed on the second page, is used to obtain a prediction probability for an upcoming patient. Supplementary Table 1: laboratory data obtained on admission of all patients. Supplementary Table 2: demographics and clinical characteristics of patients in the training and validation set. Supplementary Table 3: demographics and clinical characteristics of patients in test set. Supplementary Table 4: type and combination of organ failure in different sets of patients. Supplementary Table 5: the input features for feature selection by using K-fold cross validation. Supplementary Table 6: the predictive performance by single optimal feature in all candidate feature subset of six models. [file 5525118.f1.zip › 5525118.f1.doc]

Supplementary table 1. Laboratory data obtained on admission of all patients

| **Features** | | |
| --- | --- | --- |
| **Complete blood cell count** | | |
| white blood cell (WBC) | neutrophil count (NEUT) | hematocrit (HCT) |
| platelet count (PLT)  platelet-large cell ratio (P-LCR) | mean platelet volume (MPV)   plateletocrit (PCT) | platelet distribution width (PDW) |
| **Coagulogram** | | |
| prothrombin time (PT) | activated partial thromboplastin time (APTT) | Fibrinogen (FIB) |
| thrombin time (TT) | d-dimer | international normalized ratio (INR) |
| **Thrombelastogram (TEG)** | | |
| Reaction time (R-time) | Kinetic time (K-time) | Alpha angle (α angle) |
| Percent fibrinolysis at 30 min (LY30) | Maximum amplitude (MA) | Coagulation index (CI) |
| **Inflammatory markers** |  |  |
| c-reactive protein (CRP)  **Liver function**  alanine aminotransferase (ALT)  gamma-glutamyl transpeptidase (GGT)  high-density lipoprotein (HDL) | procalcitonin (PCT)  aspartate aminotransferase (AST)  triglyceride (TG)  low density lipoprotein (LDL) | Interleukin-6 (IL-6)  alkaline phosphatase (ALP)  total cholesterol (TC) |
| **Renal function** | | |
| blood urea nitrogen (BUN)  **Biochemical indexes**   potassium ion (K^+^)  **Pancreatic enzyme**  amylopsin (AMY) | creatinine (Cr)  calcium ion (Ca^2+^)  lipase (LIP) | sodium ion (Na^+^) |
